# Retracted: Contrast and Synthetic Multiexposure Fusion for Image Enhancement

**DOI:** 10.1155/2022/9818302

**Published:** 2022-06-14

**Authors:** Computational Intelligence and Neuroscience

*Computational Intelligence and Neuroscience* has retracted the article titled “Contrast and Synthetic Multiexposure Fusion for Image Enhancement” [1], due to concerns regarding ownership of the data.

The journal was contacted by Mr. Ahmed Zubair in October 2021, who raised concerns that they owned the data presented in the article. The journal received a copy of a thesis authored by Mr. Zubair [2] along with time-stamped versions of the article on GitHub [3] and Overleaf. The thesis and versions of the article prior to the publication of the article contain substantially overlapping figures, methods, and results and could all be verified as being created earlier than both the submission of the article to the journal and any documentation the listed author was able to provide.

The listed author was asked to explain how this may have occurred; however, documents received were not consistent with the proven timeline, and their response did not satisfy the editorial board. The article is therefore retracted with the agreement of the editorial board due to these concerns.

The listed author does not agree to the retraction and the notice.
